# Intestinal parasitic infections and associated factors among people living with HIV/AIDS in Ethiopia: A systematic review and meta-analysis

**DOI:** 10.1371/journal.pone.0244887

**Published:** 2020-12-31

**Authors:** Adam Wondmieneh, Getnet Gedefaw, Birhan Alemnew, Addisu Getie, Melaku Bimerew, Asmamaw Demis

**Affiliations:** 1 Department of Nursing, College of Health Sciences, Woldia University, Woldia, Ethiopia; 2 Department of Midwifery, College of Health Sciences, Woldia University, Woldia, Ethiopia; 3 Department of Medical Laboratory Sciences, College of Health Sciences, Woldia University, Woldia, Ethiopia; National Institute for Infectious Diseases Lazzaro Spallanzani-IRCCS, ITALY

## Abstract

**Background:**

Intestinal parasitic infections are major public health problems throughout the world, particularly in people living with HIV/AIDS. People living with HIV/AIDS are vulnerable groups for a variety of diseases, hence they are easily affected by opportunistic and non-opportunistic intestinal parasites due to the weakening of their immunity. Therefore, this study aimed to estimate the pooled prevalence and factors associated with intestinal parasitic infections among people living with HIV/AIDS in Ethiopia.

**Methods:**

Articles were identified through search engines in the online electronic databases PubMed/MEDLINE, EMBASE, HINARI, CINAHL, Cochrane Library, Google Scholar, and reference lists of previous studies following the PRISMA Protocol. Studies conducted between 2003 and 2020 with English language were included in this study. This review included papers with having high-quality NOS scores. Meta-analysis was computed using STATA version 11 software. Heterogeneity was assessed using the Cochrane Q-test and I^2^ test statistics. Subgroup and sensitivity analysis was employed with evidence of heterogeneity. Publication bias was determined using the funnel plot and Egger’s regression test statistic.

**Results:**

This study included a total of twenty-two cross-sectional studies with 5,833 study participants. The mean age of the study participants was 35 years old. The pooled prevalence of intestinal parasitic infection among people living with HIV/AIDS in Ethiopia was 39.15% (95%CI: 32.34, 45.95). The pooled prevalence of intestinal parasitic infections among people living with HIV/AIDS who had taking ART and who had not to start ART was found to be 28.27% (95%CI 22.47, 34.06) and 41.63% (95%CI: 28.75, 54.52) respectively. Unavailability of latrine (AOR: 4.87, (95% CI: 2.39, 9.92)), CD4+ T cell count <200cells/μl ((AOR: 3.53, (95%CI: 1.98, 6.27)), and having a history of diarrhea (AOR: 4.79 (95%CI: 1.53, 14.99) were factors significantly associated with intestinal parasitic infections.

**Conclusion:**

In this study, the overall pooled prevalence of intestinal parasitic infections among HIV/AIDS patients in Ethiopia was relatively high. CD4+ T-cell count <200cells/μl, unavailability of a latrine, and history of diarrhea were significantly associated with intestinal parasitic infections. Therefore, the policymakers and health care professionals could give special attention to the presence of latrines, early detection and treatment of intestinal parasitic infections, and early initiation of ART drugs.

## Background

Intestinal parasitic infections are the most common infections of human beings worldwide. Nowadays, the burden of intestinal parasitic infections (IPIs) is estimated to be around three billion of the world population [1]. Intestinal parasitic infections are major causes of morbidity and mortality worldwide, especially in low and middle-income countries. In Sub-Saharan Africa, the prevalence of intestinal parasitic infections is high [2]. Opportunistic intestinal parasitic infections such as *Cryptosporidium species* and *Isospora belli* have been reported in individuals living with human immunodeficiency virus (HIV)/ acquired immunodeficiency syndrome (AIDS) [3, 4]. Non-opportunistic parasitic infections such as; *Entamoebahistolytica*, *Giardia lamblia*, and *Ascarislumbricoides* are the commonest parasitic infections in individuals living with HIV/AIDS in low and middle-income countries [5].

According to the UNAIDS report, 37.9 million people living with HIV/AIDS worldwide. In eastern and southern Africa, approximately 20.6 million people were living with HIV/AIDS [6]. Ethiopia is highly affected by the HIV epidemic. According to the 2016 Ethiopian Demographic and Health Survey (EDHS) report, the overall prevalence of HIV/AIDS among adults in Ethiopia was 0.9% [7]. Different literature showed that diarrhea occurs in 30%-60% of AIDS patients in developed countries, whereas in low and middle-income countries about 90% of AIDS patients are affected by diarrhea, and intestinal parasitic infections are the major causes of diarrhea [8, 9].

Studies conducted in different continents showed that the magnitude of intestinal parasitic infections among HIV/AIDS patients has been varied across countries. Besides, the prevalence of intestinal parasitic infections among HIV/AIDS patients was 17% in France [10], 40% in Brazil [11], 48.8% in Iran [12], and 69% in Mexico [13]. In Africa, the magnitude of IPIs among HIV/AIDS patients was 57.48% in Cameroon [14], 24.7% in Nigeria [9], 65.3% in Burkina Faso [3], and 50.9% in Kenya [15]. Furthermore, in Ethiopia, intestinal parasitic infection among HIV/AIDS patients was 24.6% in Aksum [16], 29.1% in Gondar [17], 30.6% in Bahir Dar [18], and 33.79% in Harar [19].

Intestinal parasitic infections have a significant economic burden worldwide, especially in low and middle-income countries where settings of educational, economical, and trained manpower problems have existed. In Ethiopia, intestinal parasitic infection among people living with HIV/AIDS is estimated to be high due to low levels of personal and environmental hygiene, contamination of food and water, and improper disposal of human and animal excreta [20, 21]. Identifications of contributing factors are important to decrease the incidence of intestinal parasitic infections and to develop appropriate evidence-based guidelines and strategies.

Despite different single studies reporting the prevalence of IPIs and its associated factors, there are no national studies that show the nationwide IPIs burden towards HIV/AIDS patients in Ethiopia. Therefore, this systematic review and meta-analysis aimed to estimate the pooled prevalence of IPIs and associated factors among people living with HIV/AIDS in Ethiopia.

## Methods and materials

### Study protocol and registration

This systematic review and meta-analysis were reported based on ‘the Preferred Reporting Items for Systematic Review and Meta-analysis’ (PRISMA) guidelines [22]. The completed PRISMA checklist is provided as a supplementary file (S1 Table). The protocol for this systematic review and meta-analysis was registered on the International Prospective Register of Systematic Reviews (PROSPERO) database. The registration number is CRD42020152270.

### Search strategy and databases

This systematic review and meta-analysis was carried out using published and unpublished studies searched from electronic databases such as PubMed/MEDLINE, HINARI, EMBASE, CINAHL, Cochrane Library, and Google Scholar, and Ethiopian university repository. We also manually searched the gray literature and other related studies to identify additional relevant articles. Studies reporting the magnitude of intestinal parasitic infection among people living with HIV/AIDS and/or factors associated with intestinal parasitic infection among HIV/AIDS patients in Ethiopia were included in the final analysis. The search was conducted using the following keywords and phrases separately or in combination like “prevalence”, “magnitude”, “burden”, “intestinal parasitic infection”, “opportunistic intestinal parasitic infections”, “associated factors”, “contributing factors”, “risk factors”, “HIV/AIDS”, and “Ethiopia”. The search strings were developed using “AND” and “OR” Boolean operators. Furthermore, a reference list of included studies was further screened to identify additional relevant articles.

### Study selection criteria

#### Inclusion criteria

Studies only conducted in Ethiopia and meeting the following inclusion criteria were included in this systematic review and meta-analysis.

*Study design*. All published and unpublished observational studies (cross-sectional, case-control, and cohort studies) were included.

*Language*. Only studies reported in the English language were included.

*Study period*. Articles published from 1^st^ January 2000 to 1^st^ November 2020 were included in this study.

*Study participants*. Adult patients living with HIV/AIDS were included.

*Measurement of outcome*. Studies reported the prevalence of intestinal parasitic infections among people living with HIV/AIDS and/or predictors were considered in this study and reported a quality control methods.

#### Exclusion criteria

Studies reported in non-English language, case reports, trials, qualitative studies, reviews, policy and program evaluations, letters, abstracts, conference proceedings, and news were excluded from this study. Additionally, articles that were not fully accessed at the time of the search (full text not available, not responding to contacting the corresponding author through email two times) were excluded from this systematic review and meta-analysis.

### Outcome of measurement

This systematic review and meta-analysis had two main outcomes. The primary outcome was the pooled prevalence of intestinal parasitic infections among HIV/AIDS patients in Ethiopia, whereas, predictors of intestinal parasitic infections among people living with HIV/AIDS patients in Ethiopia were the second outcome. The association between intestinal parasitic infections and associated factors was assessed in terms of the odds ratio.

#### Intestinal parasitic infections

These are gastrointestinal tract infections caused by one or more parasites [2].

### Data extraction

After the systematic search was completed, all articles retrieved from all databases were imported into Endnote reference software version 8, and duplicates were removed. Data were extracted using a data extraction format, which was adapted from the Joanna Briggs Institute (JBI) data extraction format [23]. Three investigators (AW, AD, and GG) independently evaluated the individual study title and abstracts using predetermined article selection criteria. Three authors (AW, AD, GG) also assessed the full text based on eligibility criteria and included in the final analysis. A disagreement between investigators was resolved through discussion. Finally, all included studies were extracted using a standardized data extraction format prepared in Microsoft Excel sheets by three authors (AW, AD, and GG). The data extraction format included the first author name, year of publication, the region of the study conducted, sample size, study design, and prevalence of IPIs, and quality of each study.

### Quality assessment

In this systematic review and meta-analysis, the quality was assessed using the Newcastle-Ottawa Scale (NOS) for cross-sectional study quality assessment tool [24]. The methodological quality of each study (sampling strategy, response rate, and representativeness of the study), comparability, and outcome were checked using the NOS tool. Studies with a score of ≥ 7 out of 10 were considered as achieving good quality. This cut-off point was declared after reviewing relevant kinds of literature. Three authors (AW, AD, and GG) independently assessed the quality of each original study using the quality assessment tool. The disagreements between the two authors were resolved through discussion with a third reviewer (GG). In this study, all articles were included because they scored more than seven and above in the NOS quality assessment criteria.

### Data processing and analysis

The extracted data were imported to STATA (Corporation, College Station, Texas) version 11 software for analysis. The meta-analysis was computed using a random-effects meta-analysis model with 95% CI. Heterogeneity within the included studies was assessed using Cochrane’s Q statistics (Chi-square) and I^2^(%) with its corresponding p values [25, 26]. In this meta-analysis, the results of statistical analysis showed that there was high heterogeneity across the included studies (I^2^ = 97%, P <0.001). For the evidence of marked heterogeneity within the included studies, subgroup analysis was computed to identify the possible source of heterogeneity based on (sample size, publication year, and study region), and sensitivity analysis was computed to see the level of heterogeneity. The presence of potential publication bias was determined using the Egger’s test and presented graphically by a funnel plot [27]. The estimated pooled prevalence was reported with a 95% confidence interval (CI) and P values <0.05 were considered statistically significant. The odds ratio was computed to show the strength of the association between the intestinal parasites among HIV/AIDS patients and its predictors.

### Ethics approval and consent to participate

Not applicable.

## Results

### Selection and identification of studies

**Fig 1:**
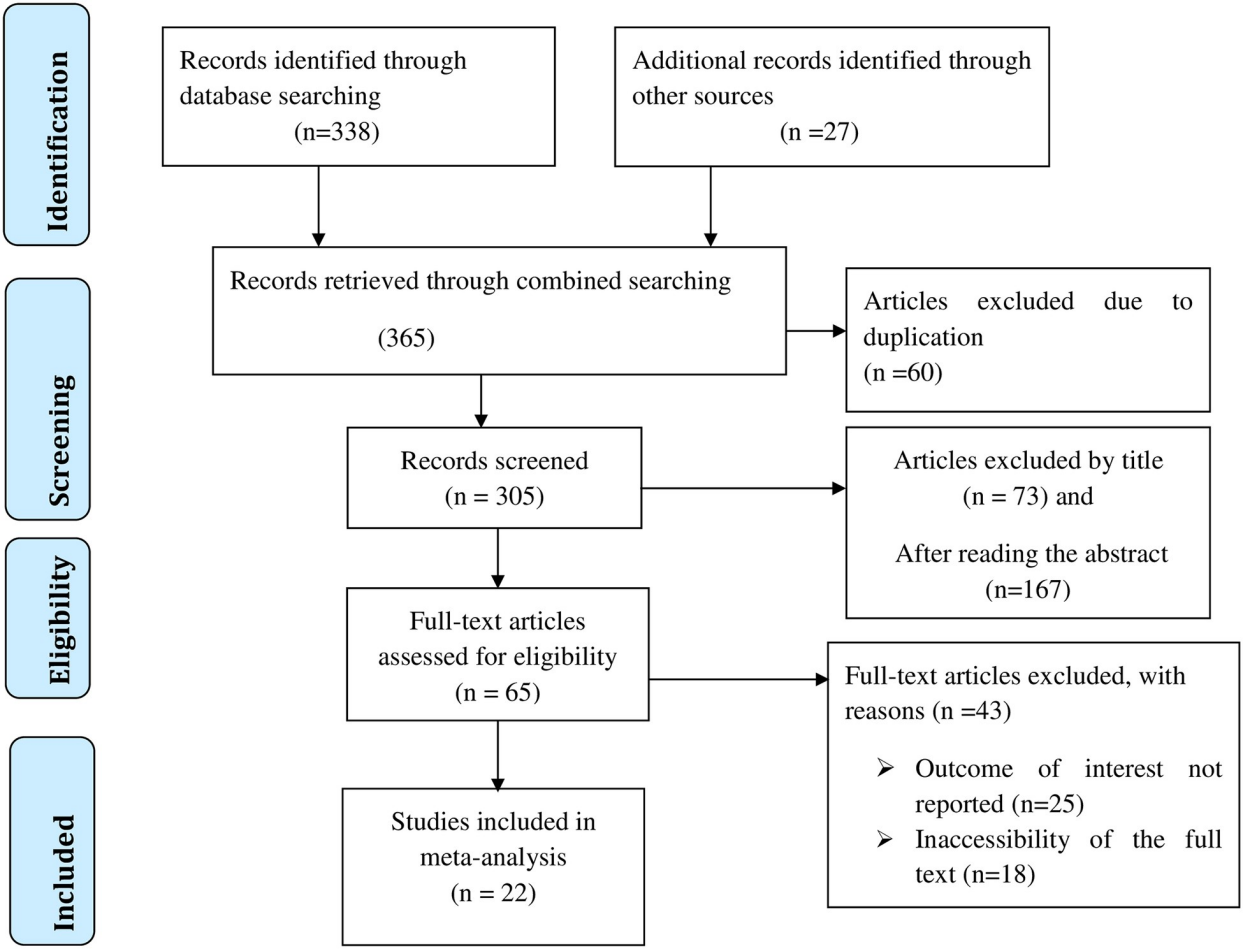
Flow chart of study selection for systematic review and meta-analysis on the prevalence and associated factors of intestinal parasitic infections among people living with HIV/AIDS in Ethiopia.

As illustrated in Fig 1, we searched and identified 365 articles regarding the prevalence and associated factors of intestinal parasitic infections among peoples living with HIV/AIDS in Ethiopia. After removing 60 duplicates, a total of 305 studies were retrieved of which 73 were rejected just by reading only the titles and 167 were excluded after reading their abstracts. Hence, 65 studies were accessed and assessed for eligibility based on the inclusion and exclusion criteria. Finally, 22 studies fulfilled the eligibility criteria and were included in the final systematic review and meta-analysis (Fig 1).

### Characteristics of included studies

Nineteen cross-sectional studies with a total of 5,833 study participants were included in this systematic review and meta-analysis. More than half of the study participants were female (58.16%). The mean age of the study participants was 35 years old. Nearly one-third of the study participants (32.54%) had not started ART. The lowest and highest sample size of the included studies was 91 [28] and 491 [29], respectively. The lowest prevalence of IPIs among HIV/AIDS patients was observed in a study conducted in Kombolcha health center, Amhara region (13.9%) [30], and the highest magnitude of IPIs was observed in a study conducted at Bahirdar town Gambi clinic (80.3%) [4]. In this study, 22 cross-sectional articles were reported from one city administration and six regions of Ethiopia. Eight studies conducted in Amhara regional state [4, 17, 18, 30–34], six studies were done in Oromia regional state [28, 35–39], four studies conducted in South Nations, Nationalities and Peoples Regional state (SNNPR) [5, 29, 40, 41], two articles reported from Tigray regional state [16, 21], one study reported from Harari regional state [19] and one study was conducted in Oromia region, Afar region and Dire Dawa city administration [42] (Table 1).

**Table 1:** Baseline characteristics of cross-sectional studies included in the meta-analysis of intestinal parasitic infection among HIV/AISD patients in Ethiopia.

No.	Author	Publication Year	Region	Sample size	Prevalence %	Quality
1	Alemu et al [4]	2011	Amhara	188	80.3	7
2	Missaye et al [43]	2013	Amhara	272	28.3	7
3	Kiros et al [18]	2015	Amhara	399	30.6	8
4	Eshetu et al [17]	2017	Amhara	223	29.1	7
5	Gebretsadik et al [30]	2018	Amhara	223	13.9	7
6	Alemayehu et al [34]	2020	Amhara	383	25.3	8
7	Gietaneh et al [33]	2019	Amhara	380	24.2	7
8	Gebrecherkos et al [32]	2019	Amhara	150	45.3	8
9	Gedle et al [40]	2017	SNNP	323	35.9	8
10	Alemu et al [41]	2018	SNNP	220	28.18	7
11	Shimelis et al [29]	2016	SNNP	491	35.8	8
12	Fekadu et al [5]	2013	SNNP	343	47.8	7
13	Adamu et al [44]	2013	Oromia	378	61.9	8
14	A. Zeynudin et al [28]	2013	Oromia	91	39.56	7
15	T.Mariam et al [38]	2008	Oromia	109	65.12	7
16	Kindie et al [37]	2016	Oromia	120	45	7
17	Awol et al [35]	2003	Oromia	192	44.8	7
18	Mesfun et al [39]	2019	Oromia	163	18.4	7
19	Mahmud et al [21]	2014	Tigray	372	56	7
20	Gebrewahid et al [16]	2019	Tigray	242	26.4	8
21	Teklemariam et al [19]	2013	Harer	371	33.7	7
22	Adamu et al [42]	2009	Oromia, Diredawa, Afar	200	48	7

SNNP = South Nation Nationalities and People

### Prevalence of intestinal parasitic infections among HIV/AIDS patients in Ethiopia

**Fig 2:**
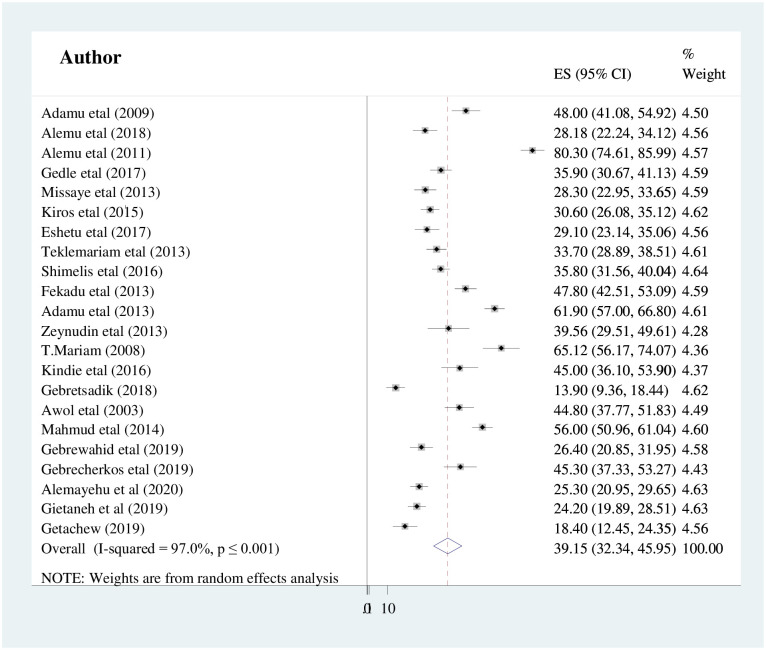
Forest plot showing the pooled prevalence of intestinal parasitic infection among people living with HIV/AIDS in Ethiopia.

The overall pooled prevalence of intestinal parasitic infections among HIV/AIDS patients in Ethiopia was found to be 39.15% (95%CI: 32.34, 45.95) (Fig 2). The Cochran’s Q statistical test and I^2^ statistics showed marked heterogeneity between the included studies (I^2^ = 97%, p<0.001). Therefore, the analysis was computed using the Der Simonian-Laired random-effects meta-analysis model. The pooled prevalence of intestinal parasitic infections among HIV/AIDS patients who had taken ART and who had not started ART was estimated to be 28.27% (95%CI 22.47, 34.06) and 41.63% (95%CI: 28.75, 54.52) respectively.

### Publication bias

**Fig 3:**
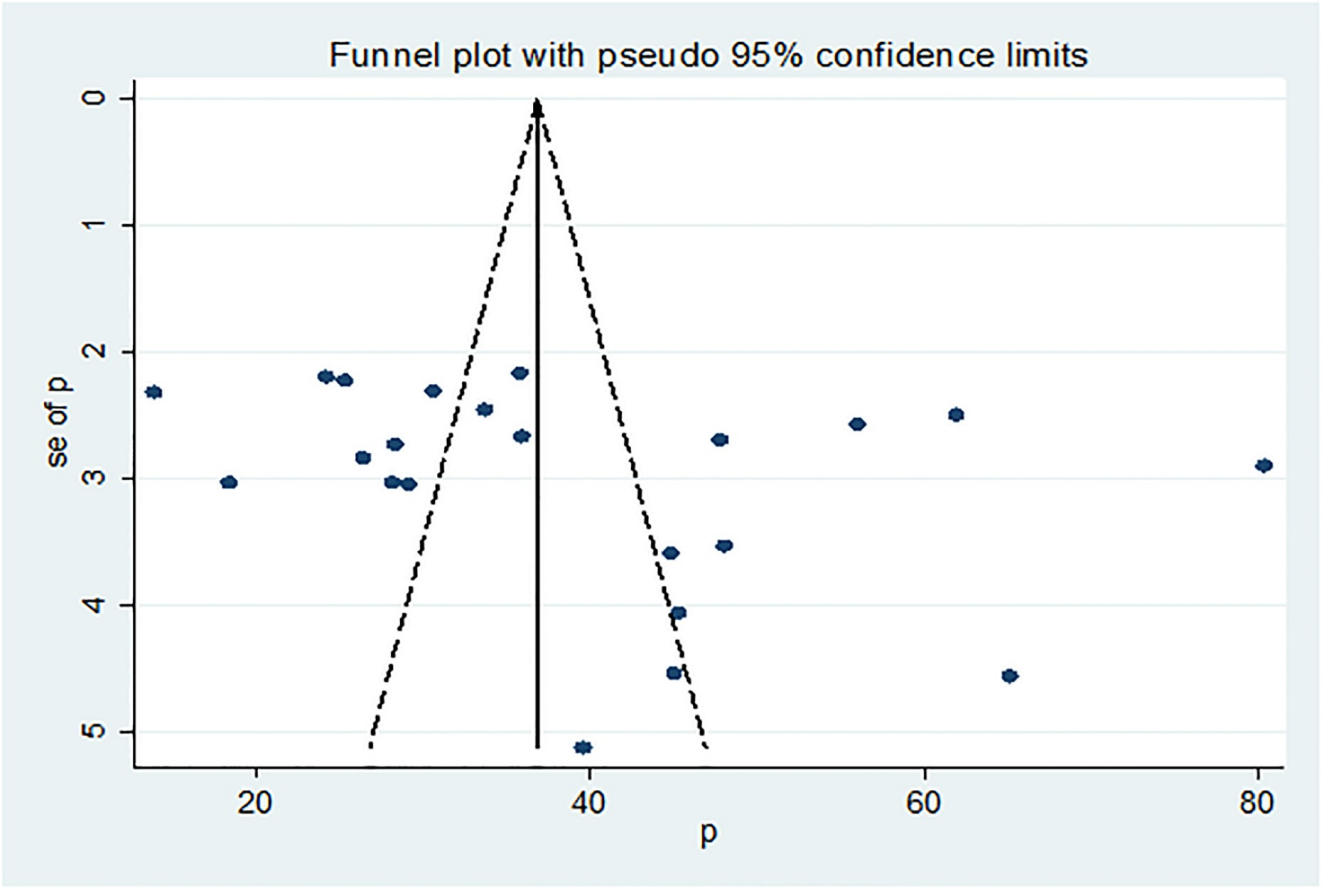
Funnel plots to test the publication bias of the included studies.

In this meta-analysis, the funnel plot showed there was a symmetrical distribution of the included studies (Fig 3). Statistically, Egger’s weighted regression test showed there was no statistically significant publication bias (p = 0.159).

### Subgroup analysis

In the current study, we computed a subgroup analysis with evidence of high heterogeneity. Hence, Cochran’s Q statistics and I^2^ statistics (I^2^ = 97%, P <0.001) showed evidence of high heterogeneity across the included studies. Therefore, subgroup analysis was computed by considering the region of the study done, sample size, and publication year to assess the possible source of heterogeneity. Hence, the highest prevalence of IPIs was observed from a study reported from Oromia region, Afar region, and Dire Dawa city administration, which accounted for 46.09% (95% CI: 33.01,59.17) followed by Tigray and Harari region accounting for 38.72% (95% CI: 21.46, 55.98), whereas the lowest prevalence of IPIs was observed in Amhara regional state with the prevalence of 34.54% (95%CI: 21.39,47.70). On the other hand, the result of subgroup analysis based on sample size showed that the prevalence of IPIs was higher in studies having a sample size of ≤300, 52.73% (95% CI: 28.35,50.27) compared to those studies having a sample size of >300, 39.15% (95% CI: 32.34,45.95). Additionally, subgroup analyses were also performed based on publication year and the results showed that IPIs was higher in studies published between 2000 to 2015, 48.70% (95% CI: 38.75,58.62) than in studies published from 2016 to 2020, 39.15% (95% CI: 32.34, 45.95) (Table 2).

**Table 2:** Subgroup pooled the prevalence of intestinal parasitic infection among HIV/AIDS patients in Ethiopia (n = 19).

Variables	Sub-groups	Included studies	Sample size	Prevalence(%) (95% CI)	I^2^, P-value
Region	Amhara	8	2,218	34.54(21.39,47.70)	98.1%, <0.001
	Oromia, Afar, Dire Dawa	7	1,253	46.09(33.01,59.17)	95.8%, <0.001
	SNNPR	4	1,377	36.98(29.59,44.37)	88.0%, <0.001
	Tigray and Harari	3	985	38.72(21.46,55.98)	97.1%,<0.001
Sample size	>300	9	3,440	39.15(32.34,45.95)	96.7%, <0.001
	≤300	13	2,393	52.73(28.35,50.27)	97.4%,<0.001
Publication year	2000–2015	11	2915	48.70(38.75,58.62)	96.9%, <0.001
	2016–2020	11	2918	39.15(32.34,45.95)	90.5%, <0.001
Overall		22	5833	41.79(34.36,49.22)	97%, <0.001

### Sensitivity analysis

Furthermore, sensitivity analyses were computed to evaluate whether the exclusion of any single study altered the pooled prevalence of statistical results of the summary estimate. However, none of the studies influenced the summary of pooled estimates after the step-by-step sensitivity analyses procedure performed (Table 3).

**Table 3:** Sensitivity analysis of IPIs among HIV/AIDS patients in Ethiopia.

Studies omitted	Prevalence(%) with 95% CI
Adamu et al [42]	36.49 (35.31, 37.68)
Alemu et al [41]	37.17 (35.98, 38.63)
Alemu et al [4]	34.90 (33.71, 36.09)
Gedle et al [40]	36.87 (35.67, 38.07)
Missaye et al [43]	37.25 (36.05, 38.45)
Kiros et al [18]	37.27 (36.06, 38.48)
Eshetu et al [17]	37.13 (35.94, 38.32)
Teklemariam et al [19]	37.02 (35.81, 38.22)
Shimelis et al [29]	36.09 (35.69, 38.12)
Fekadu et al [5]	36.26 (35.06, 37.46)
Adamu et al [44]	35.31 (34.10, 36.51)
Zeynudin et al [28]	36.78 (35.61, 37.96)
T.Mariam et al [38]	36.33 (35.15, 37.51)
Kindie et al [37]	36.68 (35.50, 37.86)
Gebretsadik et al [30]	38.45 (37.24, 39.66)
Awol et al [35]	36.59 (35.41, 37.78)
Mahmud et al [21]	35.73 (34.53, 36.93)
Gebrewahid et al [16]	37.31 (36.11, 38.50)
Gebrecherkos et al [32]	36.63 (35.45, 37.82)
Alemayehu et al [34]	37.72 (36.50, 38.93)
Gietaneh et al [33]	37.83 (36.61, 39.04)
Mesfun et al [39]	37.56 (36.32, 38.75)
Overall	39.15 (32.34, 45.95)

### Common intestinal parasitic infections among HIV/AIDS patients

According to the current meta-analysis, the overall pooled prevalence of common types of intestinal parasites among HIV/AIDS patients was observed from 22 studies. *E*. *histolytica/dispar* 12.48% (95% CI: 9.31, 15.60), *Cryptosporidium spp* 7.16% (95% CI: 4.85, 9.47) and *G*. *lamblia* 6.04% (95% CI: 4.46, 7.62) were the most common intestinal parasites detected in HIV/AIDS patients in Ethiopia with its respective I-squared percentage and p-value as showed in Table 4.

**Table 4:** Pooled prevalence of common intestinal parasites among HIV/AIDS patients in Ethiopia.

Type of intestinal parasite	Pool prevalence 95%CI	I-Squared and p-value
*E*. *histolytica/dispar*	12.48 (9.31, 15.60)	94.3%, p<0.001
*Cryptosporidium spp*	7.16 (4.85, 9.47)	94.0%, p<0.001
*G*. *lamblia*	6.04 (4.46, 7.62)	87.1%, p<0.001
*A*. *lumbricoides*	5.21 (3.62, 6.79)	92.2%, p<0.001
*Isospora belli*	1.87 (1.06, 2.68)	77.8%, p<0.001

### Factors associated with intestinal parasitic infections among HIV/AIDS patients in Ethiopia

**Fig 4:**
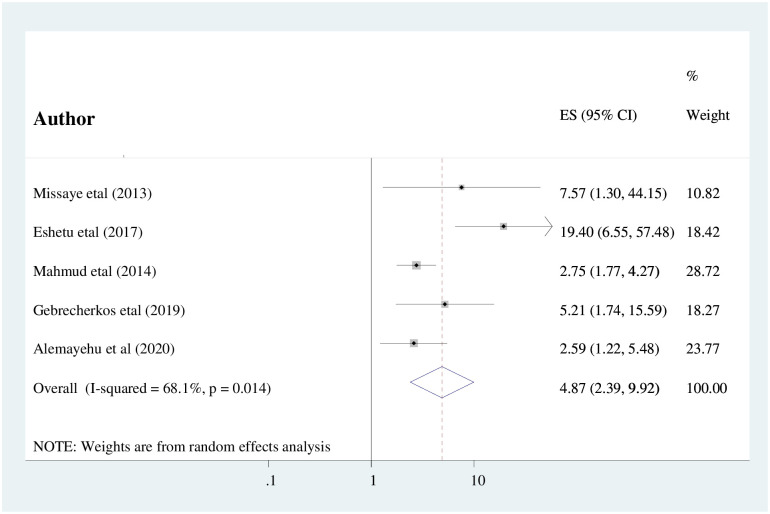
The pooled odds ratio of the association between availability of latrine and IPIs among people living with HIV/AIDS in Ethiopia.

The findings of this meta-analysis revealed that there was a significant statistical association between the unavailability of latrines and intestinal parasitic infection among people living with HIV/AIDS in Ethiopia. Five cross-sectional studies were assessed to determine the association between the unavailability of latrines and intestinal parasitic infections among HIV/AIDS patients [17, 21, 32, 43, 45]. The results showed that the risk of intestinal parasitic infection occurrence was 4.8 times higher among HIV/AIDS patients who did not have latrine as compared to those who had access to the latrine (AOR: 4.87, (95% CI: 2.39, 9.92)). Substantial heterogeneity was detected within the studies (I^2^ = 68.1% and P = 0.014). Therefore, the random-effects meta-analysis model was computed. The absence of publication bias was declared using Egger’s test statistics with a p-value of 0.31 (Fig 4).

**Fig 5:**
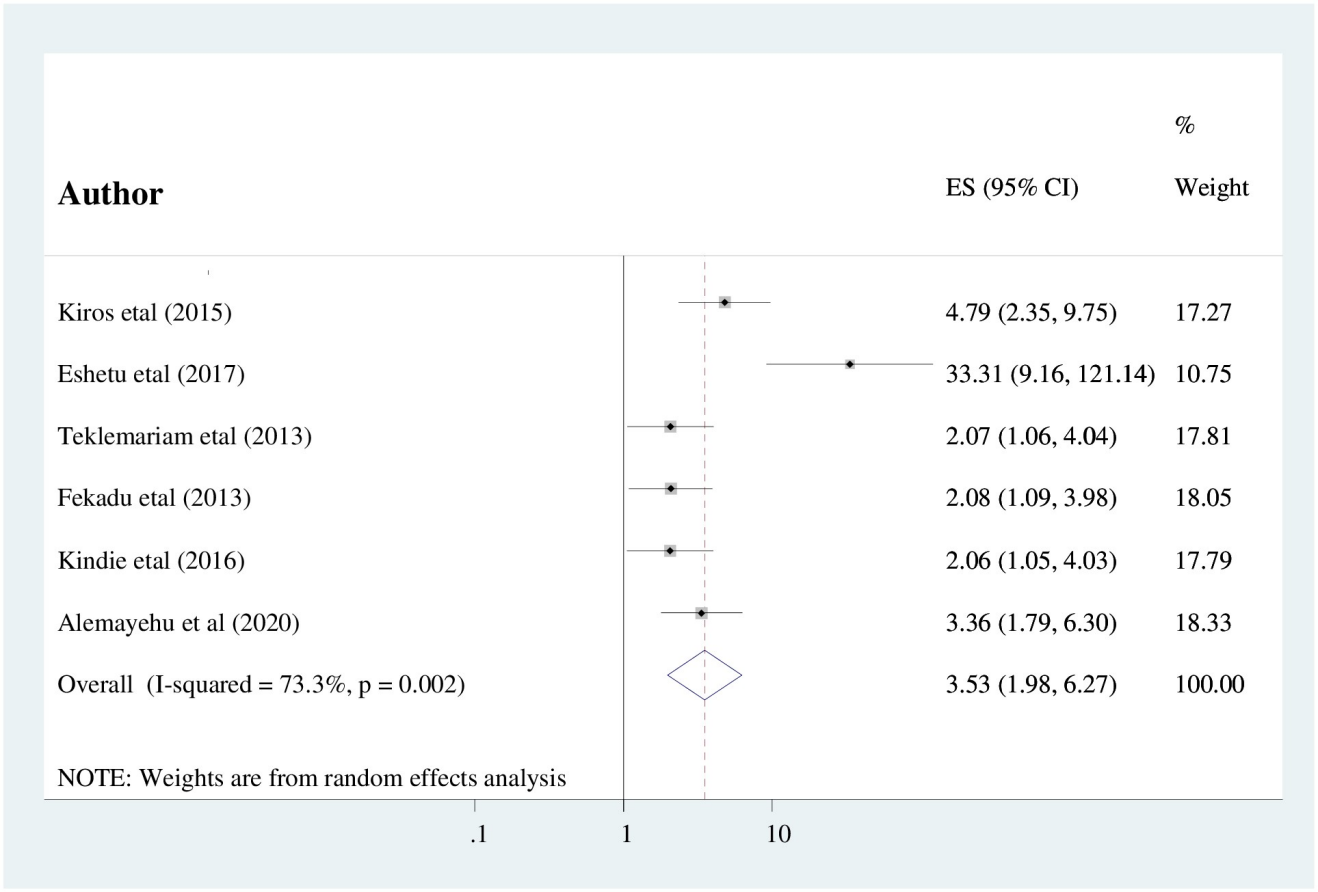
The pooled odds ratio of the association between CD4+ T-cell count and intestinal parasitic infection among people living with HIV/AIDS in Ethiopia.

The association between CD4+ T-cell counts <200cells/μl and intestinal parasitic infection among HIV/AIDS patients was computed using six studies [5, 18, 19, 37, 39, 45]. The findings of this meta-analysis showed that HIV/AIDS patients with CD4+ T-cell count <200cells/μl were statistically significant as compared to their counterparts (AOR: 3.53, (95%CI: 1.98, 6.27)) (Fig 5). Substantial heterogeneity (I^2^ = 73.2%; P = <0.001) was detected within the included studies. Therefore, the random-effects meta-analysis model was computed. Furthermore, the absence of publication bias was evidenced using Egger’s test statistics with a p-value of 0.064.

**Fig 6:**
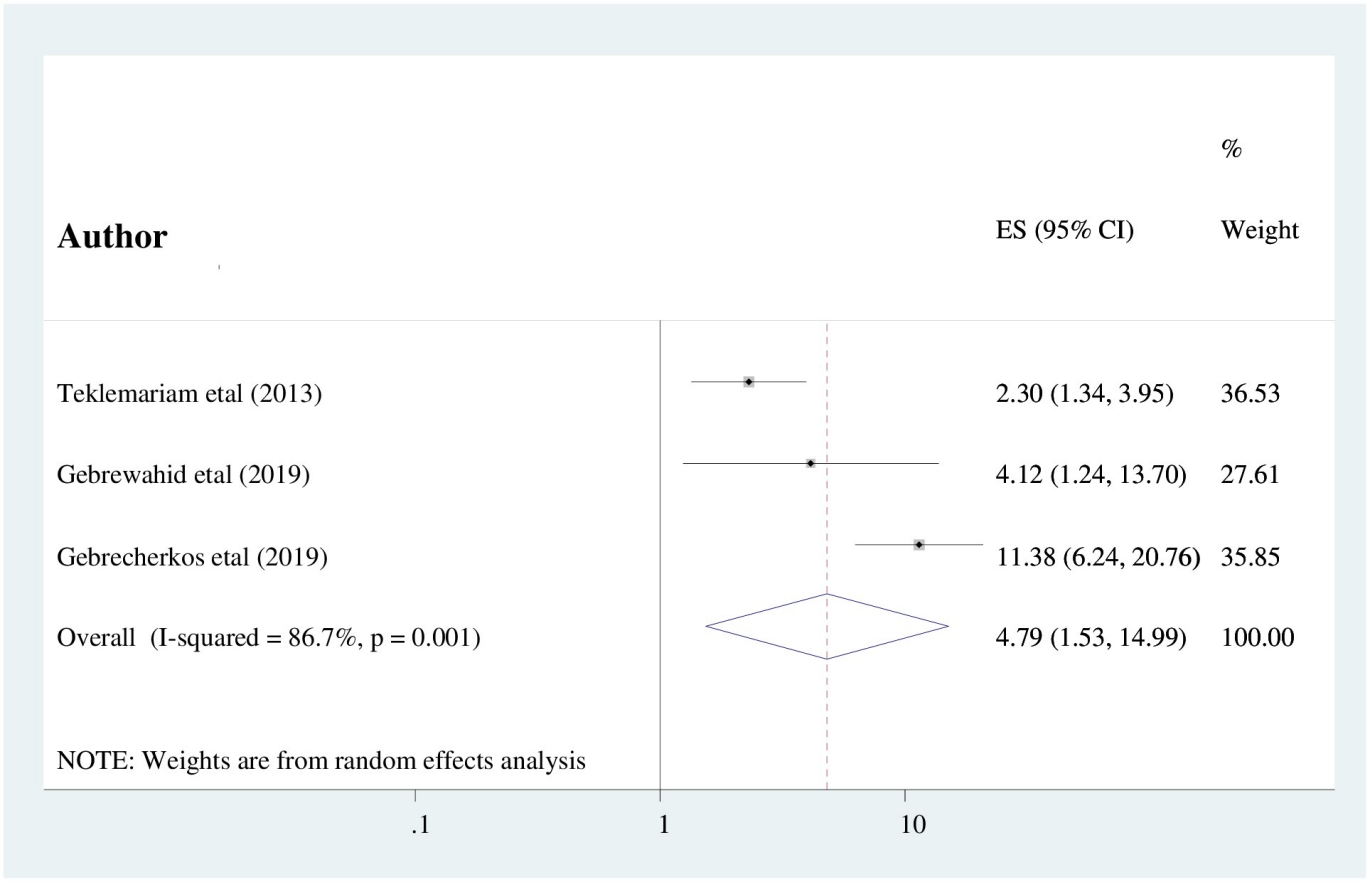
The pooled odds ratio of the association between complaint of diarrhea and IPIs among people living with HIV/AIDS in Ethiopia.

Finally, the association between diarrhea and intestinal parasitic infections among HIV/AIDS patients within the included three studies has been examined and computed [16, 19, 32]. The pooled results of this study showed that HIV/AIDS patients who had a history of diarrhea were statistically significant as compared to their counterparts (AOR: 4.79 (95%CI: 1.53, 14.99)) (Fig 6). There was evidence of considerable heterogeneity across the included studies (I^2^ = 86.7%; P ≤0.001). Therefore, the meta-analysis was computed using the random effects meta-analysis model. Furthermore, the absence of publication bias was detected using Egger’s test statistics with a p-value of 0.941.

## Discussion

The intestinal parasitic infection continues the global burden of people living in low and middle-income countries. The current systematic review and meta-analysis aimed to assess the pooled prevalence of intestinal parasitic infections and its predictors among peoples living with HIV/AIDS in Ethiopia.

In the current findings, the overall pooled prevalence of intestinal parasitic infections among people living with HIV/AIDS in Ethiopia was found to be 39.15% (95%CI: 32.34, 45.95). Even although there was no analogous meta-analysis study conducted on the specific research question. The result of this study is higher than a systematic review and meta-analysis of intestinal parasitic infections among the general population in Ethiopia (25.01%) [46]. Additionally, this finding is higher than a systematic review and meta-analysis of intestinal parasitic infections among food handlers working in Ethiopian university cafeterias (28.5%) [45]. This variation might be due to the fact that HIV infection weakens the immune system of individuals and hence they are easily affected by opportunistic and non-opportunistic intestinal parasites compared with the general population.

Besides, this study finding is lower than the study done in African countries such as in Burkina Faso (65.3%) [3], Cameroon (57.48%) [14], and Kenya (50.9%) [47]. On the contrary, the finding of this study is higher than the study done in Nigeria (20.9%) [48] and Ghana (25.2%) [49]. The possible reason for this variation might be due to differences in socioeconomic status, behavioral and socio-cultural beliefs and practices, and geographical location of study participants as well as the methodological differences (eligibility of study participants and the quality of the studies, and the number of study participants).

The current finding is in line with a study conducted in Brazil (40%) [11]. The result of this study is lower than a study done in Mexico (69%) [13] and Iran (48.8%). On the contrary, the finding of this study is higher than a study done in France (17%) [10]. This is due to the fact that in Ethiopia there is poor socioeconomic status, inaccessibility of latrines and clean water, poor quality, and low coverage of health services might contribute to this high magnitude of intestinal parasitic infections among peoples living with HIV/AIDS. Moreover, in this study, nearly one-third of the study participants (32.54%) had not started ART, which might contribute to increasing viral load, decreasing immunity; further results in easily susceptibility to infection.

Subgroup analysis was conducted based on the study sample size, the region of the study conducted, and the publication year of the study. As a result, the highest prevalence of IPIs among HIV/AIDS was observed in those studies with their sample size being less than or equal to 200 participants with the prevalence of 52.73% (95% CI: 40.20, 65.25). The possible explanation of this difference is because when the sample size increases, which would provide the true estimate of the study.

In the current study, *E*.*histolytica/dispar* 12.48% (95% CI: 9.31, 15.60), *Cryptosporidium* 12.3% (95% CI:4.85, 9.47) and *G*. *lamblia* 6.04% (95% CI: 4.46, 7.62) were the most common intestinal parasites among people living with HIV/AIDS. Similarly, *E*.*histolytica/dispar* 14.09%, *G*. *lamblia* 10.03%, and *Cryptosporidium* 5.93% were the most common parasitic infection among the Ethiopian population [46]. Additionally, *E*.*histolytica/dispar* 6.38%, *A*.*lumbricoides* 4.12%, and *G*. *lamblia* 3.12% were the most common parasitic infection among food handlers in Ethiopia [45]. This finding is in line with studies conducted in African countries such as in Nigeria, *Cryptosporidium* (10.16%), *Ameobiasis* (11.23%), and lower than studies done in Nigeria, *Giardiasis* (10.69%) [48] and Ghana, *Giardiasis* (11.4%) [49]. Whereas the result of this study was higher than studies conducted in Ghana, *Cryptosporidium* (2.05%), *Ameobiasis* (1.17%) [49]. This variation might be due to methodological variation (difference in the number of study participants, quality of laboratory equipment, and eligibility of participants). Additionally, there is a variation in geographical location, socioeconomic status, and accessibility of health services.

This study revealed that CD4+ T-cell count <200cells/μl, unavailability of a latrine, and history of diarrhea were the significant risk factors of intestinal parasitic infections for people who are living with HIV/AIDS in Ethiopia.

The odds of having intestinal parasitic infection occurrence were nearly five times higher among HIV/AIDS patients who did not have latrine access as compared to those who had access to the latrine (AOR: 4.87, (95% CI: 2.39, 9.92)). The result of this study is congruent with a study done in Port Harcourt, Nigeria [50]. This could be due to the fact that in Sub-Saharan Africa including Ethiopia, most of the people living in the rural areas used open defecation due to inaccessibility of latrine, improper solid waste management system, and inaccessibility of clean water might contribute to the higher magnitude of intestinal parasitic infections among people living with HIV/AIDS in Ethiopia.

Having CD4+ T-cell count <200cells/μl were statistically associated with intestinal parasitic infection. The odds of having intestinal parasitic infections were 3.5 times higher among patients having CD4+ T-cell count <200cells/μl as compared with their counterparts (AOR: 3.53, (95%CI: 1.98, 6.27)). This finding is supported by the study conducted in India [51] and Southwest Cameroon [52]. The possible justification could be due to the fact that a qualitative and quantitative reduction in CD4+ T cell count due to HIV could result in increased susceptibility to opportunistic infections. A study done in Cameroon and Nepal showed that *Cryptosporidium*, *Cyclospora*, and *Isospora belli* parasites were commonly observed among patients with CD4+ cell counting less than 200cells/ μl [53, 54].

Finally, people who are living with HIV/AIDS having a history of diarrheal disease were nearly five times more likely to develop intestinal parasitic infections as compared to their counterparts (AOR: 4.79 (95%CI: 1.53, 14.99). This study finding is supported by the study conducted in France [10] and Southern Iran [12]. The possible justification might be due to the fact that diarrhea in immune-compromised patients might result in increased susceptibility to infection due to loss of fluid and electrolytes. Besides, more than 85% of Ethiopian people are living in a rural area with poor health-seeking behavior, poor sanitation due to the inaccessibility of clean water and latrines resulting in a high prevalence of intestinal parasitic infection among those who had a history of diarrheal diseases in addition to HIV infection.

## Limitations of the study

All included studies reported the health facility population and so this review does not consider home-dwelling people with HIV/AIDS. The effects of viral load suppression on intestinal parasitic infection and type of parasite among advanced HIV (CD4< 200) and PLWHA were not reported in the original study, as a result, pooling was impossible. Finally, almost all included studies were cross-sectional, which might share the nature of cross-sectional study design limitations.

## Conclusion

In this study, the overall estimated pooled prevalence of intestinal parasitic infections among HIV/AIDS patients in Ethiopia was relatively high. CD4+ T-cell count <200 cells/ul, unavailability of a latrine, and history of diarrhea were significantly associated with intestinal parasitic infection. The findings of this study should trigger policymakers and health care professionals to prevent the burden of the problem through early detection, and treatment of infections and early initiation of HAART drugs can bring about a substantial reduction in the morbidity and mortality of HIV/AIDS patients in the future. Further national population-based studies are required for a more accurate estimate of the prevalence of intestinal parasitic infections among HIV/AIDS patients in Ethiopia.

## Supporting information

S1 Table PRISMA 2009 checklist. (DOC)

S1 File Excel sheet dataset. (XLSX)

## Abbreviations

AIDS: Acquired Immune Deficiency Virus
CI: Confidence Interval
HAART: Highly Active Antiretroviral Therapy
HIV: Human Immunodeficiency Virus
IPIs: Intestinal parasitic Infections
OR: Odds Ratio
PLWHA: People Living With HIV/AIDS
SNNPR: South Nation Nationalities and Peoples Region
